# Development and Validation of a Hypoxia-Related Signature for Predicting Survival Outcomes in Patients With Bladder Cancer

**DOI:** 10.3389/fgene.2021.670384

**Published:** 2021-05-26

**Authors:** Facai Zhang, Xiaoming Wang, Yunjin Bai, Huan Hu, Yubo Yang, Jiahao Wang, Yin Tang, Honggui Ma, Dechao Feng, Dengxiong Li, Ping Han

**Affiliations:** ^1^Department of Urology, West China Hospital, Institute of Urology, Sichuan University, Chengdu, China; ^2^Department of Urology, The Affiliated Hospital of Guizhou Medical University, Guiyang, China; ^3^School of Clinical Medicine, Guizhou Medical University, Guiyang, China; ^4^The Second People’s Hospital of Yibin, Yibin, China

**Keywords:** bladder cancer, hypoxia, signature, prognosis, TCGA, GEO

## Abstract

**Objectives:**

This study aimed to develop and validate a hypoxia signature for predicting survival outcomes in patients with bladder cancer.

**Methods:**

We downloaded the RNA sequence and the clinicopathologic data of the patients with bladder cancer from The Cancer Genome Atlas (TCGA) (https://portal.gdc.cancer.gov/repository?facetTab=files) and the Gene Expression Omnibus (GEO) (https://www.ncbi.nlm.nih.gov/geo/) databases. Hypoxia genes were retrieved from the Molecular Signatures Database (https://www.gsea-msigdb.org/gsea/msigdb/index.jsp). Differentially expressed hypoxia-related genes were screened by univariate Cox regression analysis and Lasso regression analysis. Then, the selected genes constituted the hypoxia signature and were included in multivariate Cox regression to generate the risk scores. After that, we evaluate the predictive performance of this signature by multiple receiver operating characteristic (ROC) curves. The CIBERSORT tool was applied to investigate the relationship between the hypoxia signature and the immune cell infiltration, and the maftool was used to summarize and analyze the mutational data. Gene-set enrichment analysis (GSEA) was used to investigate the related signaling pathways of differentially expressed genes in both risk groups. Furthermore, we developed a model and presented it with a nomogram to predict survival outcomes in patients with bladder cancer.

**Results:**

Eight genes (AKAP12, ALDOB, CASP6, DTNA, HS3ST1, JUN, KDELR3, and STC1) were included in the hypoxia signature. The patients with higher risk scores showed worse overall survival time than the ones with lower risk scores in the training set (TCGA) and two external validation sets (GSE13507 and GSE32548). Immune infiltration analysis showed that two types of immune cells (M0 and M1 macrophages) had a significant infiltration in the high-risk group. Tumor mutation burden (TMB) analysis showed that the risk scores between the wild types and the mutation types of TP53, MUC16, RB1, and FGFR3 were significantly different. Gene-Set Enrichment Analysis (GSEA) showed that immune or cancer-associated pathways belonged to the high-risk groups and metabolism-related signal pathways were enriched into the low-risk group. Finally, we constructed a predictive model with risk score, age, and stage and validated its performance in GEO datasets.

**Conclusion:**

We successfully constructed and validated a novel hypoxia signature in bladder cancer, which could accurately predict patients’ prognosis.

## Introduction

Bladder cancer is the most common cancer in the urinary system, which ranks 11th among all diagnosed cancers and has clear male predominance (Siegel et al., 2017; Witjes et al., 2021). More than 90% of bladder cancer cases were urothelial carcinoma, and approximately 75% of patients with bladder cancer were non-muscle infiltration of bladder cancer (Babjuk et al., 2019). Although the clinical outcomes have improved with the application of minimally invasive surgery, radiotherapy, neoadjuvant chemotherapy, and immunotherapy, about 25% of cases presented with muscle invasiveness or metastasis, which was still a tough problem to be solved (Wu et al., 2020; Witjes et al., 2021). Furthermore, compared with the traditional pathological prognostic indicators, an increasing number of researchers have paid more attention to prognostic and predictive molecular biomarkers with the widespread use of the next-generation sequence. For instance, molecular subtypes of bladder cancer have been accepted widely and might be incorporated into clinical management in the future, once some prospective studies validate molecular subtypes’ efficacy (McConkey and Choi, 2018).

Hypoxia was a hallmark of the tumor microenvironment, which was caused by rapid proliferation of tumor cells and the intercapillary distance longer than that of oxygen diffusion (Gilkes et al., 2014; Petrova et al., 2018). Hypoxia and hypoxia-inducible factors could regulate the expression of multiple genes in tumor cells and influence functions of tumor cells, such as proliferation, angiogenesis, invasion, metastasis, and immune invasion (Gilkes et al., 2014; Rankin and Giaccia, 2016; Lin et al., 2020; Liu et al., 2020; Mo et al., 2020). Moreover, hypoxia in the tumor microenvironment still played significant roles in treatment resistance, including radioresistance, chemoresistance, and immunosuppression (Zou et al., 2019; Lin et al., 2020; Zhang et al., 2020). Therefore, it is helpful to search for hypoxia-related genes and to change the hypoxia microenvironment for survival prediction and treatment. Lin et al. (2018) once reported that O(2)-generating MnO(2) nanoparticles succeeded in increasing the oxygen concentration of the bladder cancer microenvironment in vitro and in vivo, which enhanced the therapeutic effect of photodynamic therapy on bladder cancer. Hypoxia markers *GLUT-1* and *CAIX* were identified as independent prognostic factors and associated with vascularity and proliferation in bladder cancer (Hoskin et al., 2003; Boström et al., 2016). Additionally, some hypoxia-related non-coding RNAs were mentioned in bladder cancer, like *miR-210*, *circELP3*, *circRNA_403658*, and *lncRNA-UCA1*, which could influence functions of tumor cells and act as therapeutic targets or predictive factors (Irlam-Jones et al., 2016; Xue et al., 2017; Su et al., 2019; Wei et al., 2019).

Although some hypoxia genes have been identified and have robust predictive performance, there are still no hypoxia signatures in bladder cancer to date. Herein, we constructed a hypoxia-related signature in TCGA datasets and investigated its performance and relationships with other clinicopathological variables in bladder cancer. Then, we validated our hypoxia signature in GSE13507 and GSE32548.

## Materials and Methods

### Data Collection

We retrieved the TCGA dataset and downloaded the RNA-seq data and corresponding clinical information of 408 bladder cancer samples, which was used as the training set for hypoxia signature. GSM13507 and GSM32548, including 165 and 131 cases of bladder cancer samples, respectively, from the GEO dataset were used as the external validation datasets. Moreover, the hypoxia-related genes (total 200 genes) were obtained from the hallmark gene sets of the Molecular Signatures Database.

### Identification of Differentially Expressed Hypoxia Genes in the TCGA Dataset

The “edgeR” package was used in the R software to screen differentially expressed genes with |log Fold-Change| ≥ 1 and False Discovery Rate (FDR) < 0.05 in the TCGA dataset. After that, the selected differentially expressed genes intersected with 200 hypoxia-related genes to obtain differentially expressed hypoxia genes in the training cohort.

### Construction and Validation of the Prognostic Related Hypoxia Signature

All differentially expressed hypoxia genes were screened by univariate Cox regression analysis, to identify prognosis-associated genes with a *P*-value < 0.05. Then, the screened prognosis-related hypoxia genes were incorporated into the Lasso regression model, in which penalties were applied to all prognosis-associated hypoxia genes for preventing the overfitting effects of the model. The penalty parameter (λ) for the model was determined by 10-fold cross-validation following the minimum criteria. After that, the selected genes constituted the hypoxia signature and could generate risk scores in the multivariate Cox regression model as the following formula:

$$\text{Risk}\,\text{score}=\sum_{i=1}^{n}c\,o\,e\,f\,f\,i\,c\,i\,e\,n\,t_{i}^{*}\,E\,X\,P\,{\left(m\,R\,N\,A\right)}_{i}$$

In the light of the risk score mean, all patients in the training set can be divided into two groups and the Kaplan–Meier method was used to analyze the survival outcomes of patients in the high-risk group and the low-risk group. Similarly, the same signature can also generate risk scores in GSM13507 and GSM32548, which can be used to validate survival outcomes in different risk groups.

### Correlation of the Hypoxia Signature With Clinical Parameters

Firstly, in the light of the clinical parameters, such as age, gender, T-stage, and AJCC stage, we stratified the patients to investigate whether survival outcomes were still significantly different between high- and low-risk groups in the training and validation datasets. Secondly, the patients were classified into different subgroups according to the clinical parameters and then compare risk scores of different subgroups in the training and validation datasets. Thirdly, the risk score and other clinical parameters in the training datasets were incorporated into univariate Cox regression and multivariate Cox regression to evaluate whether the risk score was an independent prognostic predictor, and then ROC curves were used to evaluate the predictive efficacy of the risk score and other clinicopathological parameters. Similarly, the predictive efficacy was also validated in the two validation datasets.

### Immune Infiltration Analysis, Tumor Mutational Burden Analysis, and Gene-Set Enrichment Analysis

We normalized the transcriptome data and use the CIBERSORT tool to estimate the contents of 22 human immune cells in each patient. After that, we compared the difference of infiltrating immune cells in the high- and low-risk groups. A *P*-value < 0.05 was considered statistically significant.

Then, we downloaded the tumor mutational data from TCGA and use the maftools package to analyze the mutational data in both the high- and low-risk groups. TMB was calculated with the tumor-specific mutation genes. After that, we listed the top mutational genes and compared the risk scores in the mutational- and wild-type cohorts. A *P*-value < 0.05 was considered statistically significant.

Moreover, we uploaded RNA-seq profiles to GSEA to investigate that differentially expressed gene-related signaling pathways in the high-risk group and the low-risk group. The enriched set were screened based on a FDR < 0.25 and *P* < 0.05 after 1000 permutations.

### Development and Validation of a Predictive Nomogram Based on Clinical Parameters and the Risk Score

Age, gender, AJCC stage, grade, and hypoxia-related risk score were incorporated into the univariate Cox regression analysis and multivariate Cox regression analysis. We selected the independent predictive factors with *P* < 0.05 to build the Cox regression model in the TCGA dataset, and the model was presented with a nomogram to facilitate clinical practice. AUC, Brier scores, and calibration plots were used to assess the performance of the model in 1, 3, and 5 years. The simple bootstrap strategy was used to validate the model internally, and GSE13507 was used to validate the model externally.

### Prediction of Chemotherapy Response

Bladder cancer patients’ response to chemotherapy drugs was predicted based on the public pharmacogenomics database Genomics of Drug Sensitivity in Cancer (GDSC)^1^. The chemotherapy drug sensitivity was evaluated by the half-maximal inhibitory concentration (IC50) with the “pRRophetic” package in R software. The drug sensitivity was compared in both different risk groups, and a *P*-value < 0.05 was considered statistically significant.

### Statistical Analysis

All statistical analyses were performed in the R software (Version 4.0.2)^2^ and GraphPad Prism 8. Quantitative data in two groups were compared using the Student *t*-test, and quantitative data in three or more groups were compared with one-way analysis of variance (ANOVA) or Welch’s test. *P* < 0.05 was regarded as statistically significant.

## Results

### Selection of Hypoxia-Related Genes and Construction of a Signature

**FIGURE 1:**
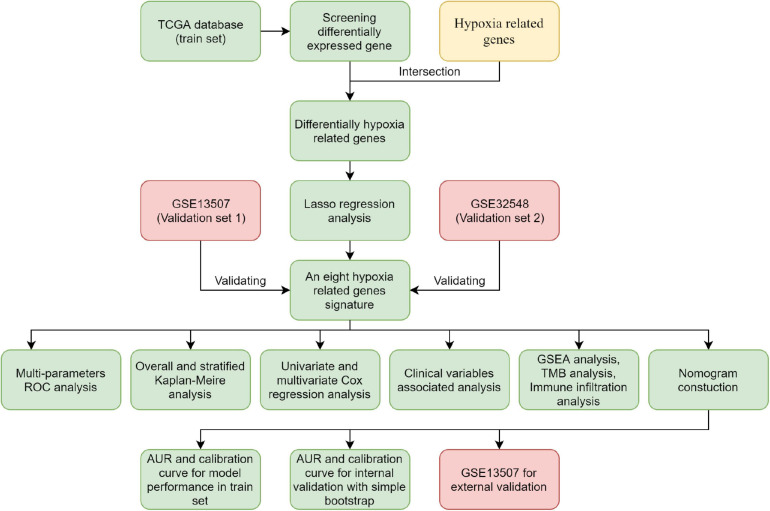
Flowchart of the analysis.

**FIGURE 2:**
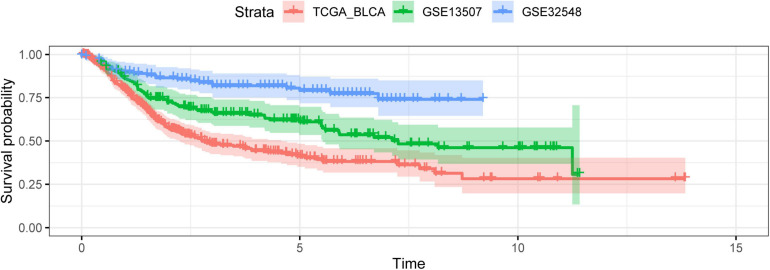
The outcome variables of three datasets presented with the Kaplan–Meier curve. The survival outcomes of patients in three cohorts were different.

**FIGURE 3:**
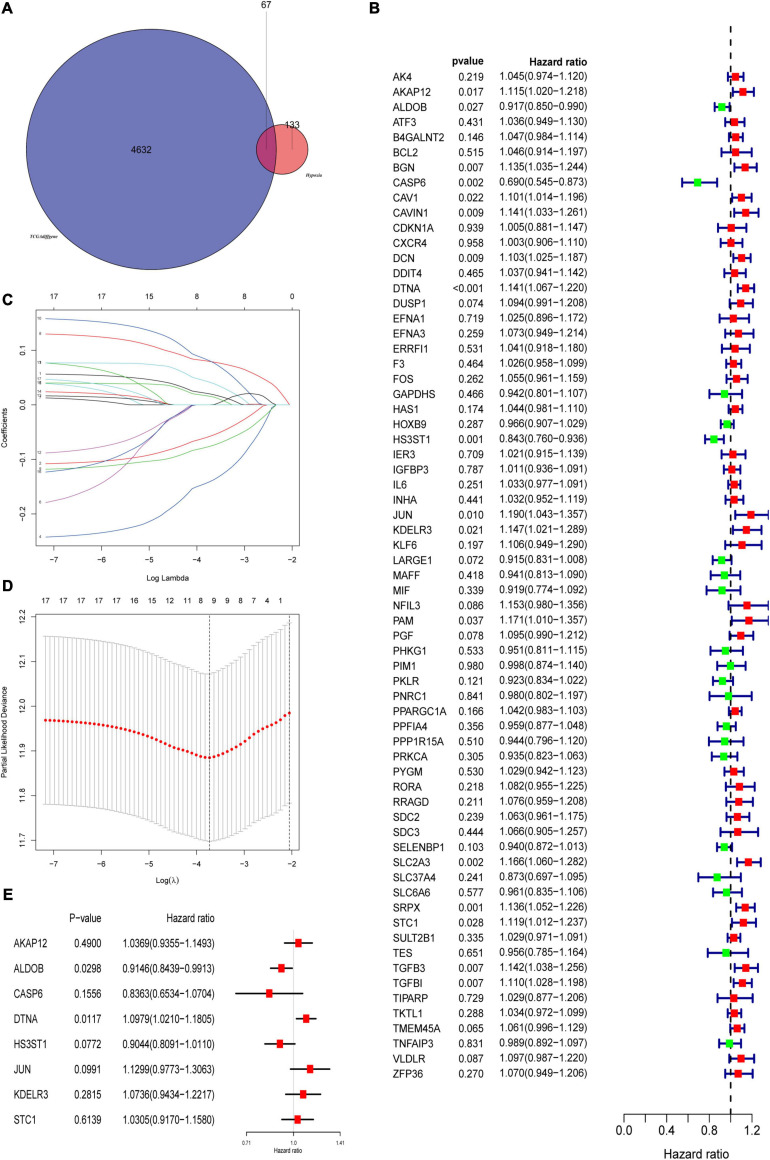
Construction of prognostic related hypoxia signature. **(A)** Differentially expressed genes intersected with hypoxia genes to obtain differentially expressed hypoxia genes in the TCGA cohort. **(B)** Univariate Cox regression analysis was used to screen prognostic related hypoxia genes. **(C,D)** The screened prognosis-related hypoxia genes were incorporated into the Lasso regression model and penalties were applied for preventing overfitting effects of the model. **(E)** The selected genes constructed the hypoxia signature and refitted the multivariate Cox regression model.

The flowchart showed the major procedures of our study (Figure 1), and the basic characteristics of these three cohorts are presented in Table 1 and Figure 2. Samples from patients without survival status or survival time were excluded in the TCGA database, and consequently 402 patients were included as our training set to construct the hypoxia signature and the subsequent predictive model. Differentially expressed genes were selected in all normalized genes, and 4,632 genes were selected with FDR < 0.05 and |log Fold-Change| ≥ 1 (Supplementary Figure 1). Then, the differentially expressed genes intersected with 200 hypoxia genes and a total of 67 differentially expressed hypoxia-related genes were selected (Figure 3A). All 67 genes were incorporated in univariate Cox regression analysis and Lasso regression analysis to identify the prognosis-associated hypoxia genes and to prevent overfitting (Figures 3B–D). Finally, eight genes (*AKAP12*, *ALDOB*, *CASP6*, *DTNA*, *HS3ST1*, *JUN*, *KDELR3*, and *STC1*) were identified and constructed the hypoxia signature. After that, we put the eight genes into the multivariable Cox regression analysis to generate risk scores according to the formula above (Figure 3E).

**TABLE 1:** Baseline clinical characteristics of three databases.

**Variables**	**Number**		
	**TCGA**	**GSE13507**	**GSE32548**
**Total**	408	165	131
**Age**			
<60	87	42	26
≥60	321	123	105
**Gender**			
Female	107	30	31
Male	301	135	100
**Grade**	10 cases missing		G1+G2 deemed as low grade
Low grade	20	105	56
High grade	378	60	75
**AJCC stage**	2 cases missing	Calculated by 7th AJCC	
0a	0	23	NA
I	2	80	NA
II	130	26	NA
III	140	29	NA
IV	134	7	NA
**T**	34 cases missing		
Tis	0	23	40
T1	3	81	53
T2	119	31	T2+T3+T4 = 38
T3	194	19	
T4	58	11	
**N**	42 cases missing		
N0	237	149	NA
N1	46	9	NA
N2	75	6	NA
N3	8	1	NA
**M**	201 cases missing		
M0	196	158	NA
M1	11	7	NA

*NA, not applicable.*

### Prognostic Value of the Hypoxia Signature in the Training Set and Validation Sets

**FIGURE 4:**
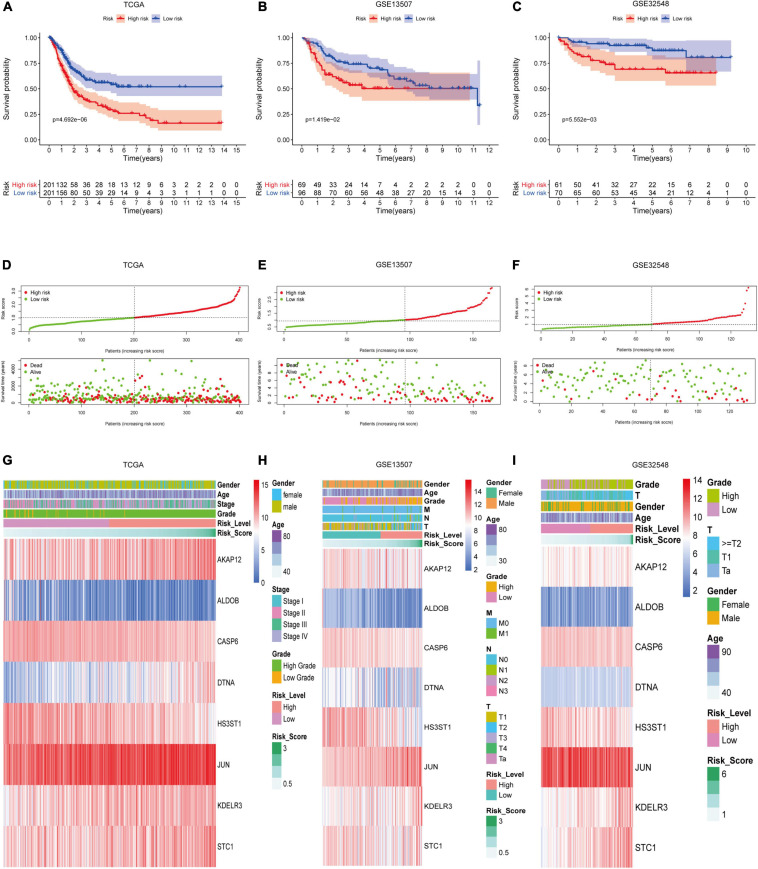
The performance of hypoxia signature in three datasets. Kaplan–Meier analysis showed that patients with lower risk scores had better overall survival than the ones with higher risk scores in the training set **(A)**, GSE13507 **(B)**, and GSE325489 **(C)**. The risk scores of patients were ranked sequentially, and patients with lower risk scores usually had longer survival time and lower mortality rates compared with those with higher risk scores in the training set **(D)**, GSE13507 **(E)**, and GSE325489 **(F)**. Multigroup heat maps of the hypoxia signature in the training set **(G)**, GSE13507 **(H)**, and GSE325489 **(I)**.

According to the mean risk score in the training set (TCGA), the patients in the three datasets were divided into the high-risk group and the low-risk group. The patients in the low-risk group had a better overall survival times than the ones in the high-risk group, which was validated in GSE13507 and GSE32548 (Figures 4A–C). As for the patients with lower risk scores, they usually had lower mortality rates and longer survival time compared with the ones with higher risk scores (Figure 4D). Similarly, the two validation sets proved this tendency as well (Figures 4E,F). Furthermore, with the increment of risk scores, the expressions of *AKAP12*, *DTNA*, *JUN*, *KDELR3*, and *STC1* increased notably in the three datasets. While the expressions of *ALDOB*, *CASP6*, and *HS3ST1* decreased obviously as risk scores increased (Figures 4G–I).

### Relationship Between the Hypoxia Signature With Clinical Parameters

**FIGURE 5:**
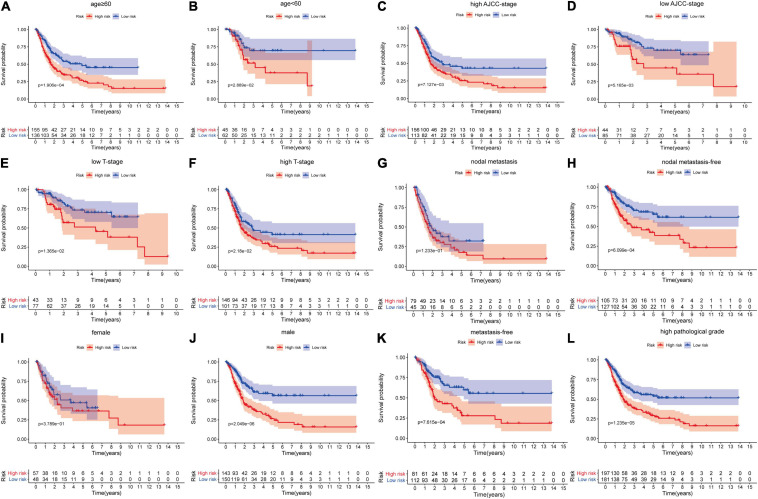
The survival outcomes of bladder cancer patients with different risk scores in subgroups of **(A)** age ≥60, **(B)** age <60, **(C)** high AJCC-stage, **(D)** low AJCC-stage, **(E)** low T stage, **(F)** high T stage, **(G)** nodal metastasis, **(H)** nodal metastasis-free, **(I)** female, **(J)** male, **(K)** metastasis-free, and **(L)** high pathological grade, in the training dataset. The patients with higher risk scores had a worse overall survival compared with those with lower risk scores in the subgroups of **(A)** age ≥60, **(B)** age <60, **(C)** high AJCC stage, **(D)** low AJCC stage, **(E)** low T stage, **(F)** high T stage, **(H)** nodal metastasis-free, **(J)** male, **(K)** metastasis-free, and **(L)** high pathological grade.

**FIGURE 6:**
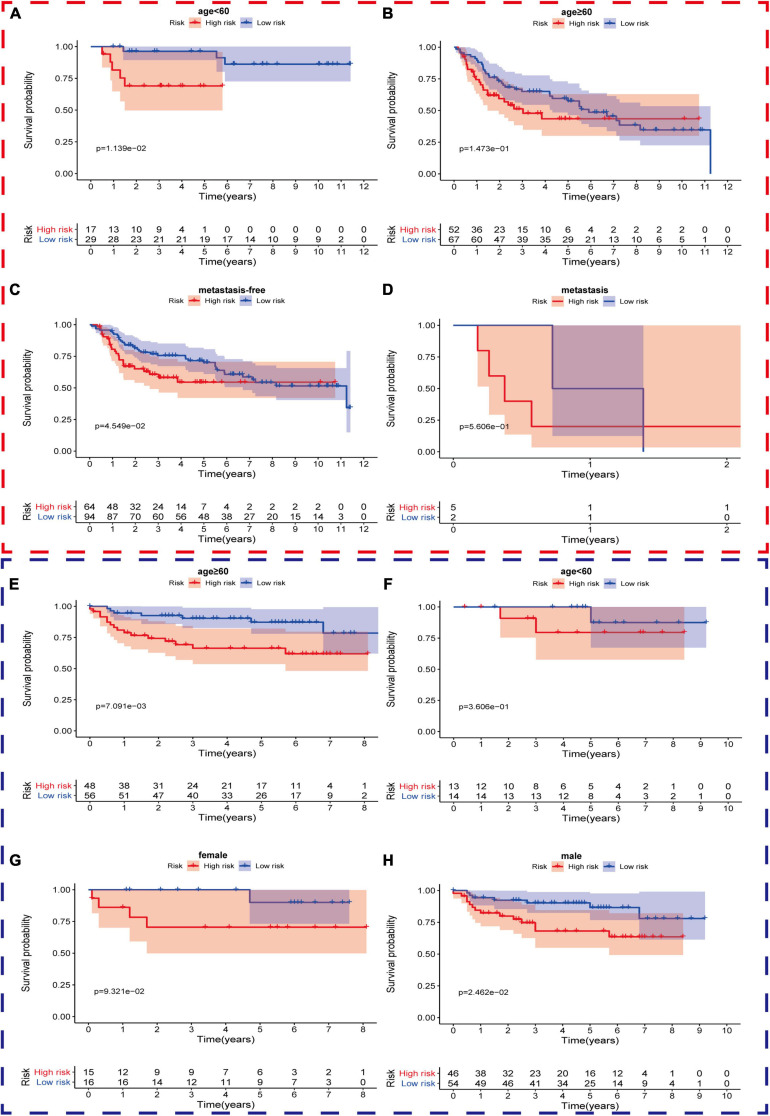
The survival outcomes of bladder cancer patients with different risk scores in subgroups of **(A)** age <60, **(B)** age ≥60, **(C)** metastasis-free, and **(D)** metastasis, in GSE13507, and subgroups of **(E)** age ≥60, **(F)** age <60, **(G)** female, and **(H)** male, in GSE32548. The red and blue dashed boxes corresponded to the subgroups in GSE13507 and GSE32548, respectively. The survival times of the low risk group were significantly longer than those of the high-risk group in the subgroup of **(A)** age ≤60 and **(C)** metastasis-free in GSE13507, while the differences of overall survival time were significantly in subgroups of **(E)** age ≥60 and **(H)** male in GSE32548.

Firstly, in order to further verify the performance of this hypoxia signature, we stratified the patients in the light of age (≥60 and <60), gender (female and male), AJCC stage (I+II and III+IV), T stage (T1–T2 and T3–T4), N stage (N0 and N1–3), M stage (M0 and M1), and pathological grade (low and high) in the training set. The Kaplan–Meier analysis showed that the patients with low risk scores had higher survival probabilities compared with the ones with high-risk scores in the subgroups of age ≥60 (*p* = 1.906e-04), age <60 (*p* = 2.889e-02), high AJCC stage (*p* = 7.127e-03), low AJCC stage (*p* = 5.165e-03), low T stage (*p* = 1.365e-02), high T stage (*p* = 2.18e-02), nodal metastasis-free (*p* = 6.099e-04), male (*p* = 2.049e-06), metastasis-free subgroup (*p* = 7.615e-04), and high pathological grade subgroup (*p* = 1.235e-05) (Figures 5A–F,H,J–L). In the same way, we also validated the signature’s performance in the subgroups stratified by clinical parameters in GSE13507 and GSE32548. The overall survival times of the low-risk group were significantly higher than those of the high-risk group in the subgroup of age ≤60 (*p* = 1.139e-02) and metastasis-free (*p* = 4.549e-02) in GSE13507, while the difference of overall survival time was significantly in subgroups of age ≥60 (*p* = 7.091e-03) and male (*p* = 2.462e-02) in GSE32548 (Figures 6A,C,E,H).

**FIGURE 7:**
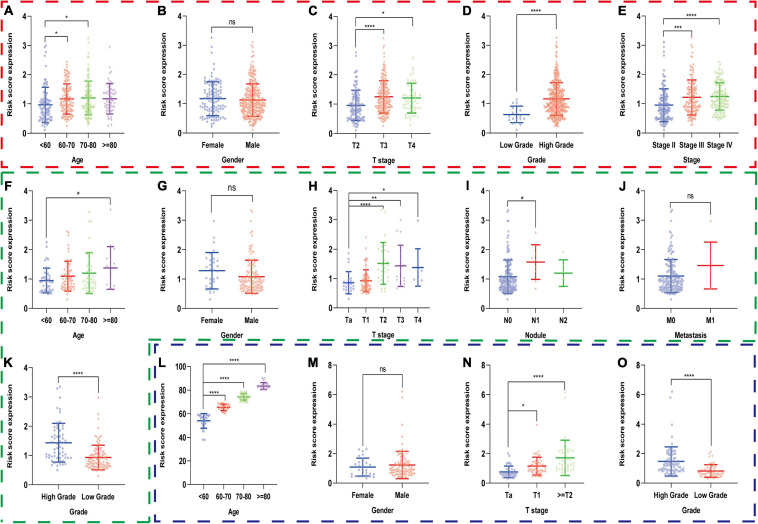
The risk scores of patients in different subgroups stratified by clinicopathologiocal parameters in the training set and two validation sets. The red, green, and blue dashed boxes corresponded to the TCGA database, GSE13507, and GSE32548, respectively. The risk scores were compared in the subgroups of **(A)** age, **(B)** gender, **(C)** T stage, **(D)** AJCC stage, and **(E)** pathological grade in the TCGA dataset, and were validated in the subgroups of **(F)** age, **(G)** gender, **(H)** T stage, **(I)** nodule, **(J)** metastasis, and **(K)** grade in GSE13507 and in the subgroups of **(L)** age, **(M)** gender **(N)** T stage, and **(O)** grade in GSE32548, respectively. *P < 0.05; **P < 0.01; ***P < 0.001; ****P < 0.0001; ns, not significance.

Secondly, we compared risk scores in different subgroups stratified by clinical parameters in the training set and found that the risk scores were significantly different in age, T stage, AJCC stage, and pathological grade (Figures 7A,C–E), which were also validated in the subgroups of age, T stage, N stage, and grade in GSE13507 and in the subgroups of age, T stage, and grade in GSE32548 (Figures 7F–O). Interestingly, we observed that the risk scores seemed not to correlate with gender, which were also validated by two GEO datasets.

**FIGURE 8:**
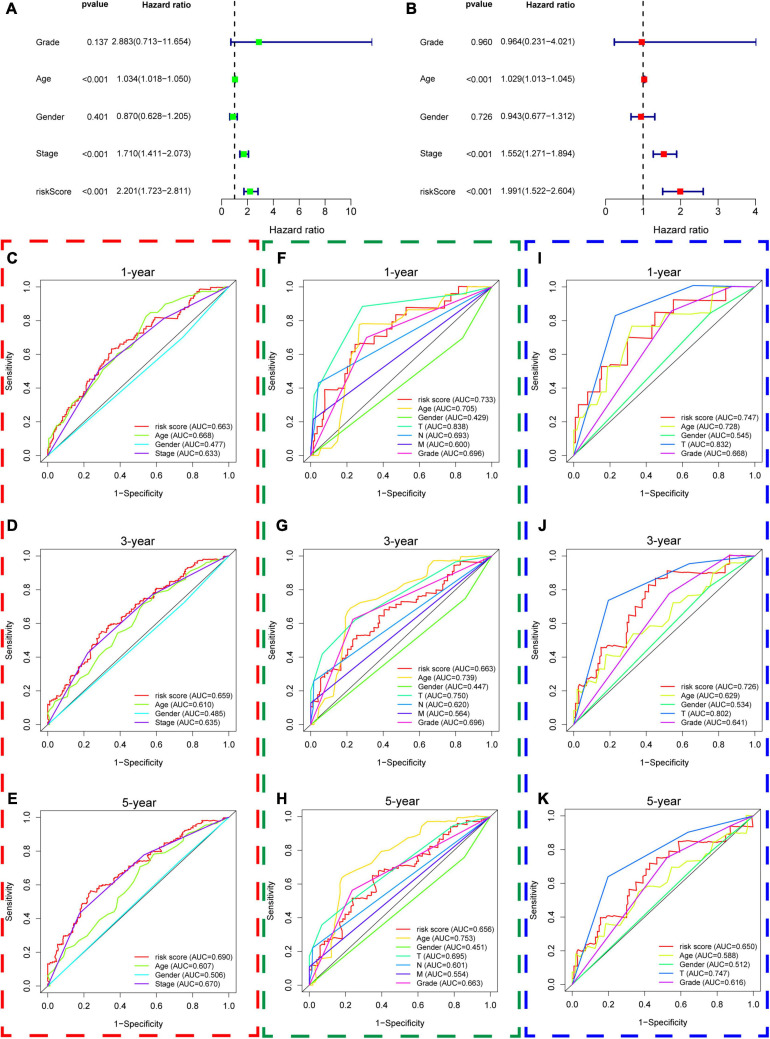
The predictive performance of the risk score and other clinicopathological parameters. **(A,B)** The univariate and multivariate Cox regression suggested that the risk score was an independent prognostic factor in the training dataset. The red, green, and blue dashed boxes corresponded to the TCGA database, GSE13507, and GSE32548, respectively. **(C–E)** Showed the 1-year multiple ROC curves of the risk score and other clinicopathological parameters in the training set, GSE13507, and GSE32548, respectively. **(F–H)** Showed the 3-year multiple ROC curves of the risk score and other clinicopathological parameters in the training set, GSE13507, and GSE32548, respectively. **(I–K)** Showed the 5-year multiple ROC curves of the risk score and other clinicopathological parameters in the training set, GSE13507, and GSE32548, respectively.

Thirdly, we put age, gender, AJCC stage, grade, and the risk score of hypoxia signature into the univariable Cox regression analysis and multivariable Cox regression analysis and identified that the risk score was a significant independent prognostic factor in the TCGA dataset (HR = 1.991 and *P* < 0.001) (Figures 8A,B). Furthermore, we used ROC curves to compare the predictive performance of different variables in 1, 3, and 5 years and found that the risk score of hypoxia signature had a robust predictive ability compared with traditional pathological parameters, which was also validated in GSE13507 and GSE32548 (Figures 8C–K).

### Relationship Between Hypoxia Signature and Immune Cell Infiltration

**FIGURE 9:**
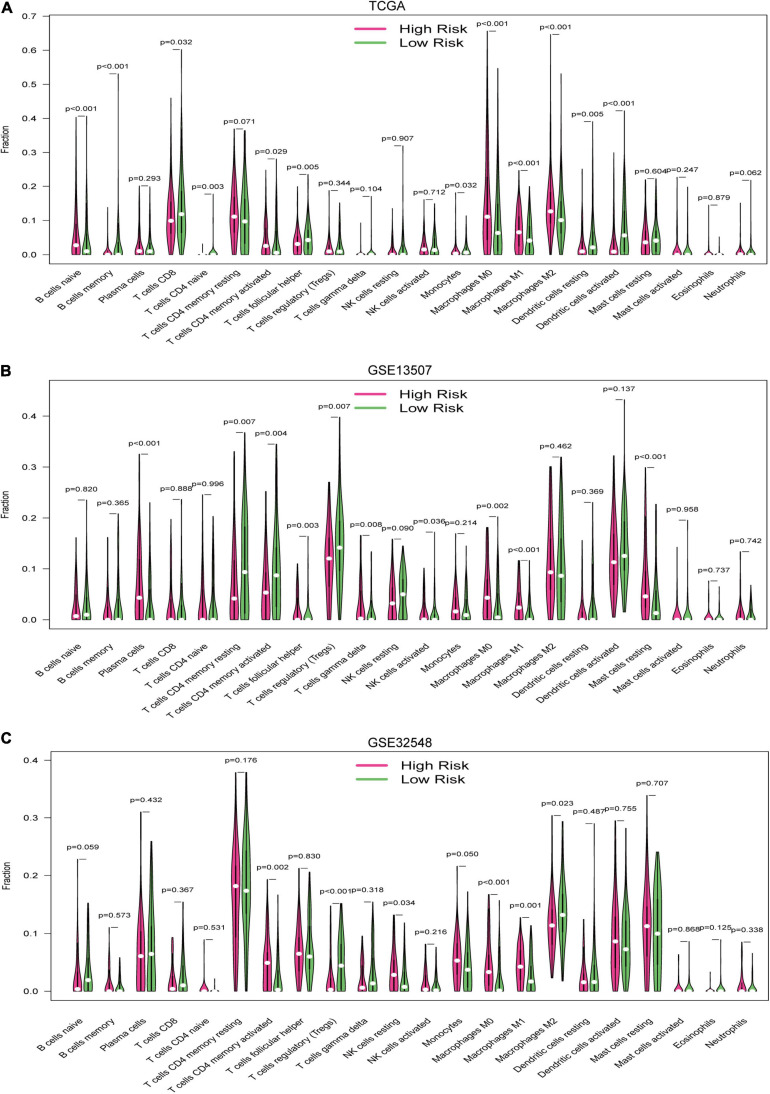
The vioplots of the content of 22 immune cells of the high-risk and low-risk groups in **(A)** the TCGA dataset, **(B)** GSE13507, and **(C)** GSE32548. Activated memory CD4+ T cells and M0 and M1 macrophages were all significantly different in these three datasets. M0 and M1 macrophages had higher infiltration in the high-risk group.

Twenty-two immune cell types were evaluated in the training set and two validation sets, and 12 immune cell types were significantly different between the high-risk group and the low-risk group in the training datasets, which were naive B cells, memory B cells, CD8^+^ T cells, naive CD4^+^ T cells, activated memory CD4^+^ T cells, follicular helper T cells, monocytes, M0 macrophages, M1 macrophages, M2 macrophages, resting dendritic cells, and activated dendritic cells (Figure 9A). Similarly, there were 10 immune cell types significantly different between both risk groups in GSE13507, which were plasma cells, resting memory CD4^+^ T cells, activated memory CD4^+^ T cells, follicular helper T cells, regulatory T cells, gamma delta T cells, activated NK cells, M0 macrophages, M1 macrophages, and resting master cells (Figure 9B). Activated memory CD4^+^ T cells, regulatory T cells, resting NK cells, M0 macrophages, M1 macrophages, and M2 macrophages were significantly different between both groups in GSE32548 (Figure 9C). Collectively, activated memory CD4^+^ T cells and M0 and M1 macrophages were all significantly different in these three datasets. M0 and M1 macrophages had a higher infiltration in the high-risk group. Unexpectedly, the difference of activated memory CD4+ T cell infiltration in both risk groups was inconsistent. In TCGA and GSE32548, the high-risk group had higher activated memory CD4+ T cell infiltration, while activated memory CD4+ T cell infiltration presented an opposite trend in GSE13507 (Figure 9).

### Tumor Mutational Burden Analysis

**FIGURE 10:**
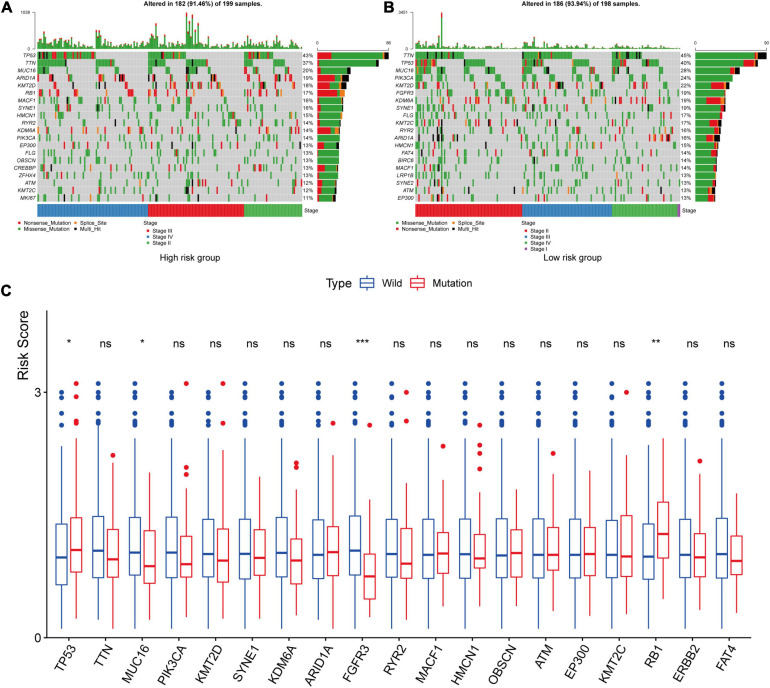
Tumor mutational burden analysis. The top 20 mutational genes were listed in the high-risk group **(A)** and low-risk group **(B)**. The risk scores between the wild type and the mutation type of the top frequent mutational genes were compared **(C)**. The asterisk on the top of the box plots meant that the risk scores were significantly different between wild and mutational types. The risk scores in the mutational types of *TP53* and *RB1* were significantly higher than those in the wild types, while the risk scores were higher in the wild types of *MUC16* and *FGFR3* compared with the mutational types. *P < 0.05; **P < 0.01; ***P < 0.001; ns, not significance.

We used the maftool package to summarize and analyze the mutational data in the TCGA datasets. In order to compare mutational genes, we listed the top 20 mutational genes in both risk groups, respectively. According to Figure 10, we could find that *TP53*, *TTN*, *MUC16*, *ARID1A*, *KMT2D*, *MACF1*, *SYNE1*, *HMCN1*, *RYR2*, *KDM6A*, *PIK3CA*, *EP3000*, *FLG*, *ATM*, and *KMT2C* were the most frequent mutational genes. In addition, *OBSCN*, *CREBBP*, *ZFHX4*, and *MKI167* belonged to the top 20 frequent mutational genes in the high-risk group, while *FGFR3*, *FAT4*, *BIRC6*, *LRP1B*, and *SYNE2* were part of the top 20 frequent mutational genes in the low-risk group. Finally, we divided the patients in the TCGA cohort according to the top frequent mutational genes status and then compared the risk scores between the wild-type and the mutation type of the top frequent mutational genes. The asterisk on the top of the box plots meant that the risk scores were significantly different between wild and mutational types (Figure 10C). Interestingly, the risk scores between the wild types and the mutation types of *TP53*, *MUC16*, *RB1*, and *FGFR3* were significantly different (Figure 10C). The risk scores in the mutational types of *TP53* and *RB1* were significantly higher than that in the wild types, while the risk scores were higher in the wild types of *MUC16* and *FGFR3* compared with the mutational types.

### Gene-Set Enrichment Analysis

**FIGURE 11:**
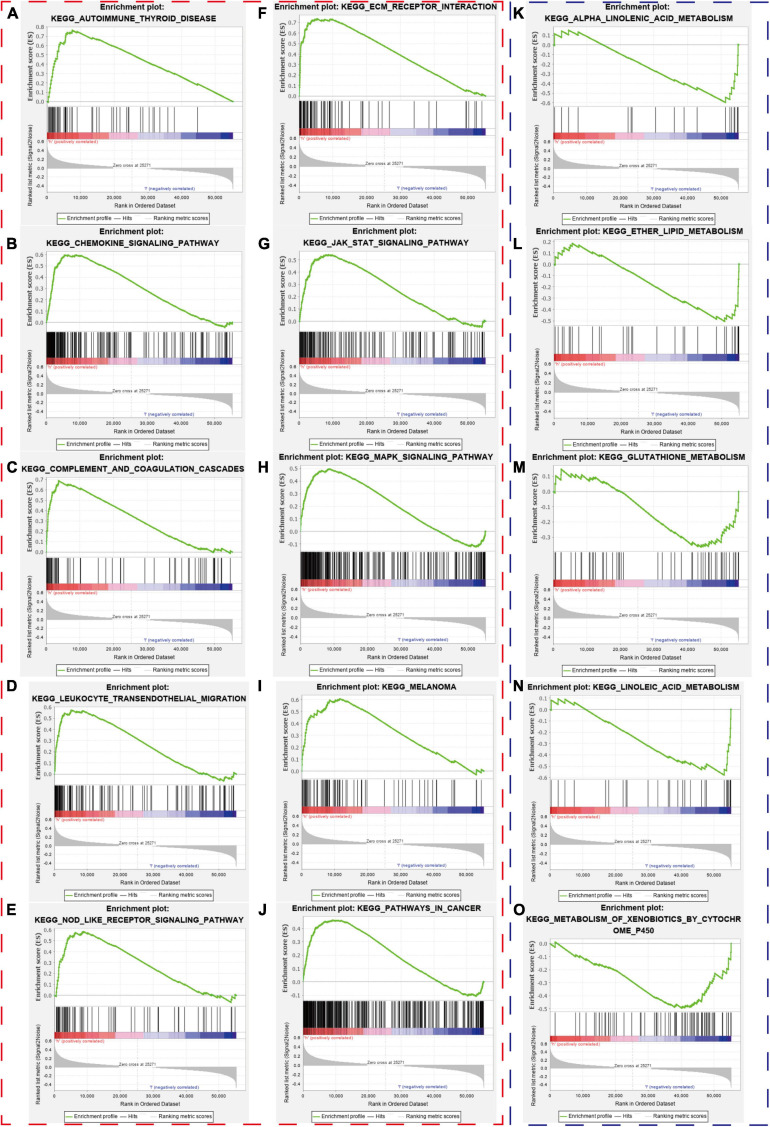
Gene-set enrichment analysis. The red and blue dashed boxes corresponded to high- and low-risk groups. GSEA results showed significant enrichment of immune- and cancer-related signaling pathways in the high-risk group **(A–J)**, and significant enrichment of metabolism signaling pathways in the low-risk group **(K–O)**.

**FIGURE 12:**
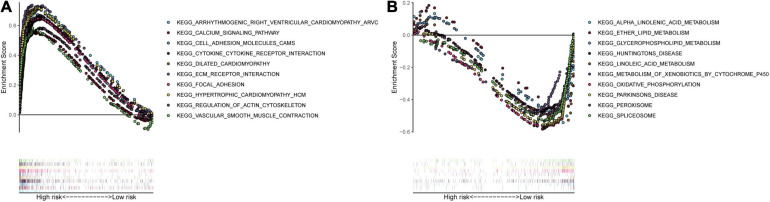
Multiple GSEA pathways in the high- and low-risk groups. The top 10 KEGG signaling pathways in the **(A)** high-risk and **(B)** low-risk groups.

We collected the top 30 KEGG molecular pathways in which different genes were enriched in both the high-risk group and the low-risk group. Interestingly, some immune or cancer-associated pathways belonged to the high-risk groups, including autoimmune thyroid disease, chemokine signal pathway, complement and coagulation cascade, leukocyte and transendothelial migration, nod-like receptor signal pathway, ECM receptor interaction, JAK-STAT signal pathway, MAPK signal pathway, melanoma, and pathway in cancer (Figures 11A–J). Moreover, some metabolism-related signal pathways were enriched into the low-risk group, including alpha linolenic acid metabolism, ether lipid metabolism, glutathione metabolism, linolenic acid metabolism, and metabolism of xenobiotics by cytochrome P450 (Figures 11K–O). Finally, we summarized the top 10 pathways in each risk group and presented them in Figure 12.

### Predictive Nomogram Construction

**FIGURE 13:**
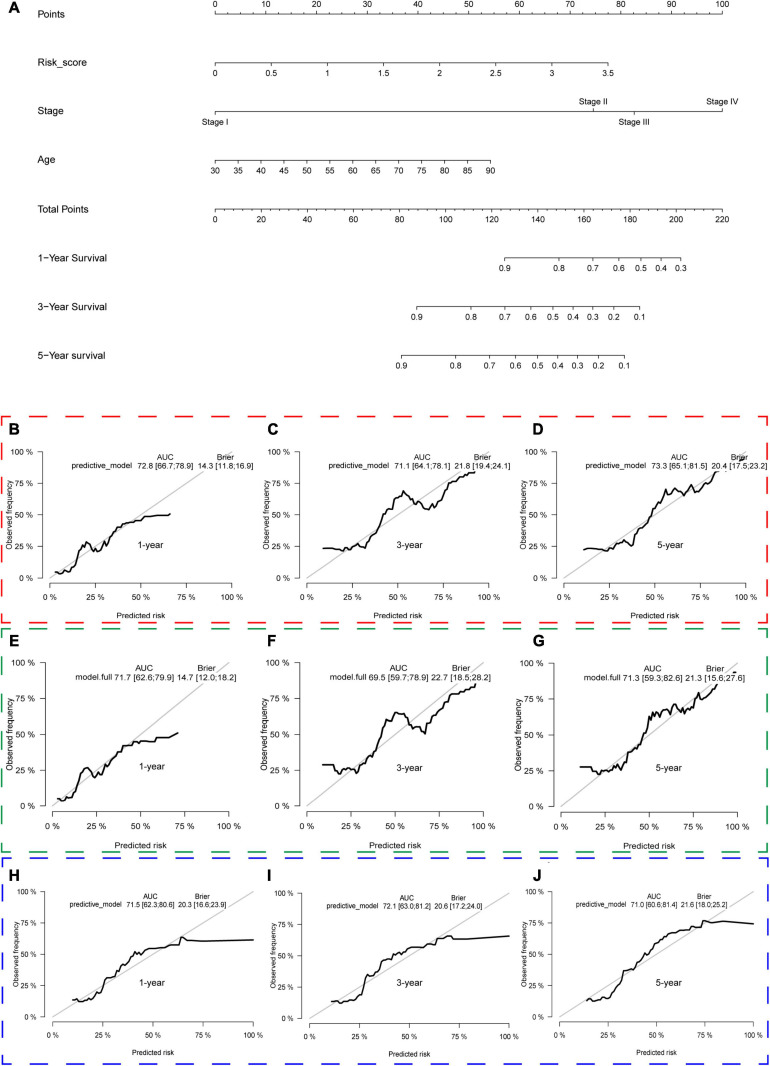
The nomogram of the gene signature and its performance in the training set and internal and external validation sets. The predictive model was presented with a nomogram **(A)**. The red, green, and blue dashed boxes corresponded to the TCGA dataset, internal validation set, and GSE13507. The AUC and Brier scores of the nomogram in 1, 3, and 5 years in the training set **(B–D)**, internal validation set **(E–G)**, and GSE13507 **(H–J)**.

We constructed a predictive model with the independent predictive factors in multivariable Cox regression analysis (Figures 8A,B), and the model was presented with a nomogram (Figure 13A). The area under ROC (AUC) and Brier scores of the predictive model in 1, 3, and 5 years were 72.8 (66.7;78.9) and 14.3 (11.8;16.9), 71.1 (64.1;78.1) and 21.8 (19.4;24.1), and 73.3 (65.1;81.5) and 20.4 (17.5;23.2) in the training set, respectively (Figures 13B–D). Similarly, we used the simple bootstrap strategy to validate the model internally, and the AUC and Brier scores in 1, 3, and 5 years were 71.7 (62.6;79.9) and 14.7 (12.0;18.2), 69.5 (59.7;78.9) and 22.7 (18.5;28.2), and 71.3 (59.3;82.6) and 21.3 (15.6;27.6) in the internal validation set, respectively (Figures 13E–G). Due to lack of AJCC stage, N stage, or M stage in GSE32548, only GSE13507 can be utilized as an external validation set to validate the nomogram. The AUC and Brier scores in 1, 3, and 5 years were 71.5 (62.3;80.6) and 20.3 (16.6;23.9), 72.1 (63.0;81.2) and 20.6 (17.2;24.0), and 71.0 (60.6;81.4) and 21.6 (18.0;25.2) in the external validation set (Figures 13H–J).

### Prediction of Chemotherapy Response

**FIGURE 14:**
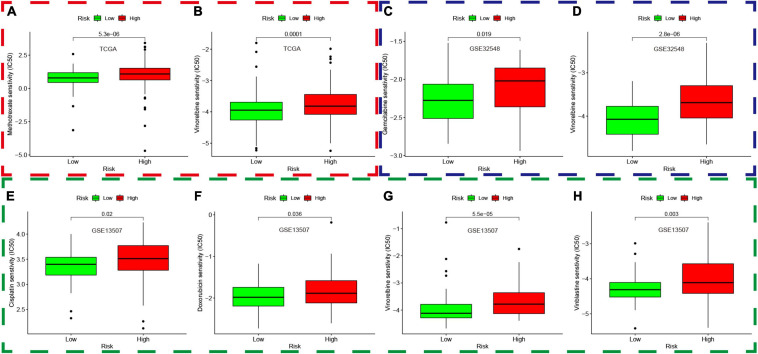
Prediction of chemotherapy response based on the genomics of drug sensitivity in cancer. The red, green, and blue dashed boxes corresponded to the TCGA database, GSE13507, and GSE32548, respectively. **(A,B)** showed that the IC50 of methotrexate and vinorelbine in the high-risk group was significantly higher than that in the low-risk group in the TCGA dataset. Similarly, the IC50 of gemcitabine and vinorelbine was significantly higher in the high-risk group compared with that in the low-risk group in GSE32548 **(C,D)**, and the IC50 of cisplatin, doxorubicin, vinblastine, and vinorelbine was significantly higher in the high-risk group in GSE13507 **(E–H)**.

The “pRRophetic” package was used to explore the data in GDSC and predict the chemotherapy response in both different risk groups in the TCGA dataset, GSE13507, and GSE32548. We selected the widely used drugs in muscle-invasive bladder cancer, such as gemcitabine (G), cisplatin (C), methotrexate (M), vinblastine (V), and doxorubicin (A), which constituted basic GC or MVAC protocol. Moreover, vinorelbine was also selected for its application in treatment of small cell carcinoma of the bladder. Interestingly, the results showed that the IC50 of methotrexate and vinorelbine in the high-risk group was significantly higher than that in the low-risk group in the TCGA dataset (Figures 14A,B), which implied that patients in the high-risk groups were more likely to develop chemoresistance. Similarly, the IC50 of gemcitabine and vinorelbine was significantly higher in the high-risk group in GSE32548 (Figures 14C,D), and the IC50 of cisplatin, doxorubicin, vinblastine, and vinorelbine was significantly higher in the high-risk group in GSE13507 (Figures 14E–H).

## Discussion

As the most common malignancy in the urinary system, bladder cancer ranks 11th among all diagnosed cancers in the world, which caused more than 16,000 deaths in the United States (Siegel et al., 2017; Babjuk et al., 2019; Witjes et al., 2021). Although neoadjuvant chemotherapy, immunotherapy, intensity-modulated radiation therapy, and minimally invasive surgeries have been applied for the treatment of bladder cancer and have improved the patients’ survival outcomes, the invasion and metastasis are still tough problems and shorten the survival times of patients with bladder cancer (Wu et al., 2020). To date, an increasing number of researchers have focused on hypoxia, a hallmark of tumor microenvironment, for it is closely related to invasion and metastasis of tumor (Gilkes et al., 2014; Rankin and Giaccia, 2016; Petrova et al., 2018). Yang et al. (2008) once reported that hypoxia and HIF-1alpha could directly regulate *TWIST*, which could promote epithelial–mesenchymal transition (EMT) and metastatic phenotypes in patients with head and neck cancer. Cox (Cox et al., 2015) found that lysyl oxidase (LOX) in the hypoxic cancer secretome could disrupt normal bone homeostasis and lead to the formation of focal pre-metastatic lesions in patients with estrogen-receptor-negative breast cancer. Furthermore, hypoxia and HIF-1alpha also promote the growth of tumor at distant sites via *VEGF-α*, which could stimulate the angiogenesis (Joyce and Pollard, 2009).

It is due to the significant roles of hypoxia that some hypoxia-related gene signatures were constructed to predict patients’ survival outcomes in the era of genomics and precision medicine. To date, the hypoxia signatures of hepatocellular carcinoma, colorectal cancer, lung adenocarcinoma, glioma, gastric cancer, and prostate cancer have been developed (Yang et al., 2018; Zou et al., 2019; Lin et al., 2020; Liu et al., 2020; Mo et al., 2020; Zhang et al., 2020). Although some hypoxia genes have been identified as biomarkers in bladder cancer, there are still no hypoxia signatures about bladder cancer. Herein, we used *AKAP12*, *ALDOB*, *CASP6*, *DTNA*, *HS3ST1*, *JUN*, *KDELR3*, and *STC1* to construct a hypoxia signature with a robust performance. The ROC of our signature in 1, 3, and 5 years were 0.663, 0.659, and 0.690, respectively, which were much higher than the ROC of the AJCC stage (Figure 8). Then, we validated our signature’s performance in GSE13507 and GSE32548, whose outcome variables and independent variables were different compared with TCGA datasets (Table 1 and Figure 2). The ROC of 1, 3, and 5 years of the hypoxia signature were still comparable with the ROC of the T stage and grade in GSE13507 and GSE32548, which illustrated the signature’s stability and wide applicability. After that, we constructed a predictive model with risk score, age, and stage in the training dataset and presented it with a nomogram. One thousand times bootstraps were used to validate the nomogram internally, and GSE13507 was utilized as an external validation set to validate the nomogram. Whatever the training dataset, the internal validation dataset, or the external validation dataset, the performance of this model is still steady and robust (Figure 13).

To deepen the understanding of the signature, we analyzed each gene in the signature. AKAP12 protein belonged to the family of kinase scaffolding protein and participated in signal transduction of cancer (Wu et al., 2018). Finger et al. (2015) observed that hypoxia-regulated AKAP12v2 could promote the invasion, migration, and metastasis of tumor via protein kinase A in melanoma. Tao et al. (2015) once reported that *ALDOB* inhibited metastasis and could serve as a prognostic biomarker in hepatocellular carcinoma, which concurred with Wang’s finding in clear cell renal carcinoma (Wang J. et al., 2019). Moreover, He et al. (2016) observed that downregulation of *ALDOB* is associated with poor prognosis of patients with gastric cancer. *CASP6* encoded Caspase-6 protein which was the executor caspase inducing apoptosis by cleaving lamin A and other substrates. Lee et al. (2006) once analyzed the entire coding region and calculated the somatic mutations in gastric carcinoma. After that, he found that loss of caspase-6 might contribute to the pathogenesis of gastric cancers. *DTNA* encoded a scaffold protein, which maintained the structural integrity of muscle cells. Liu et al. (2017) once screened key genes for early-stage colon carcinoma and found that *DTNA* had the potential diagnosis value for Colon adenocarcinoma. *HS3ST1* was involved in the biosynthesis of heparan sulfate, and Liu found that its transcriptional activity decreased significantly in glioma tissue compared with the para-tumorous tissue. In addition, the transcriptional activity in high-grade glioma tissue was lower than that in low-grade glioma (Ushakov et al., 2017). As a proto-oncogene, *Jun* was a subunit of AP-1 and participated in tumor proliferation, invasion, and metastasis. Wang et al. (2017) demonstrated that tobacco smoke elevated AP-1 activation in bladder cancer cells, and tobacco smoke-mediated cell differentiation and EMT could be reversed by AP-1 suppression. Zhao et al. (2018) reported that benzidine enhances the proliferation of bladder cells via activating the MAPK/AP-1 pathway. The researches about *KDELR3* were few. Marie et al. (2020) once conducted melanoblast transcriptome analysis and identified that the loss of *KDELR3* could impair experimental metastasis. *STC1*, a hypoxia-induced molecular target, could promote cell proliferations, invasion, migration, and metastasis in hepatocellular cancer, gastric cancer, colorectal cancer, breast cancer, and glioblastoma (Chang et al., 2015; Rezapour et al., 2016; Chan et al., 2017; Wang Y. et al., 2019; Xiong and Wang, 2019). With the exception of Jun, the remaining seven genes have not been studied in bladder cancer to date, but their ability to promote or weaken malignant phenotypes was well presented in our signature. For example, if a gene could promote the malignant phenotype, its expression was more frequent in patients with higher risk scores and vice versa (Figures 4G–I).

We explore the relationship between the hypoxia signature with clinical parameters. Firstly, we stratified the patients with some clinical parameters in the TCGA dataset and found that the patients with lower risk scores still had better survival outcomes compared with the ones with higher risk scores in the subgroups of age ≥60, age <60, male, high AJCC-stage subgroup, low AJCC-stage subgroup, low T-stage, high T-stage subgroup, nodal metastasis-free, metastasis-free subgroup, and high pathological grade (Figure 5). In addition, we also validated the signature’s prognostic performance in subgroups stratified by clinical parameters in GSE13507 and GSE32548 (Figure 6). As for the insignificant differences of survival time in the subgroups of nodal metastasis, female subgroup, low pathological grade, and metastasis, we preliminarily thought that the small sample size in the subgroups might influence the predictive ability of our signature (Figures 5G,I and Supplementary Figures 2, 3). As regards some insignificant differences of the subgroups in GSE13507 and GSE32548, we suspected that the significant different outcome variables of three datasets and small sample size in the subgroups might influence the predictive performance (Figures 2, 6). Secondly, we compared the risk scores in different subgroups stratified by clinical parameters in the training dataset and two validation datasets. It was worth noting that the subgroups with higher pathological stages or grades usually had higher risk scores, which illustrated that our molecular signature closely related to pathological parameters and had the potential to be an important prognostic indicator (Figure 7).

Immune cell infiltration in tumor tissue played a significant role in promoting or preventing the proliferation, invasion, and migration of cancer cells, so that immunotherapy had become an important treatment for tumor (Petrova et al., 2018; Liu et al., 2020). To explore the relationships between the hypoxia signature and immune cell infiltration, we compared the immune cell content of different risk score groups and found that activated memory CD4+ T cells and M0 and M1 macrophages were all significantly different in the TCGA datasets and two validation datasets. The high-risk group had higher contents of M0 and M1 macrophages compared with the low-risk group. Li (Li et al., 2020) once reported that high expression of activated CD4 (+) memory T cells and low expression of M0 macrophage were associated with better clinical prognosis in bladder cancer. Tumor-associated macrophage (TAM) played a significant role in cancer progression, metastasis, and immunotherapy resistance, which were widely infiltrated in the tumor microenvironment (Cheng et al., 2021). Macrophage plasticity allows these innate immune cells to adopt their well-known M1–M2 polarization axis (Larionova et al., 2020). TAMs with M1 profile usually expressed MHC-II, CD68, CD80, and CD86 and had important antitumor and pro-inflammation function, while the M2 microphage in the tumor microenvironment could play a role in pro-tumor and anti-inflammation (Malfitano et al., 2020; Mantovani et al., 2021). TAMs are a polarized M2 subtype of macrophage in the microenvironment of some cancers, but the concrete mechanism is not well defined. Chai reviewed 99 urothelial carcinoma cases, immunostained pathological sections, and found that higher tumor-associated macrophage (TAM) infiltration was identified in high-expression HIF-1alpha cases rather than HIF-1alpha low-expression cases. Moreover, HIF-1alpha overexpression and high TAM count were associated with worse DFS (Chai et al., 2008). Koga et al. (2004) also conducted immunochemical analysis of TAM and Endothelial Per-Arnt-Sim domain protein 1 (EPAS1) induced by hypoxia and proved that EPAS1-expressing TAM counts were significantly associated with higher T stage and progression in bladder cancer. Theoretically, M1 macrophages with the roles of antitumor and pro-inflammation should be enriched into the low-risk group with better survival, while higher contents of M1 macrophages, not M2 macrophages, were enriched into the high-risk group in our study. Delprat et al. (2020) once reported that cycling hypoxia could induce unpolarized M0 macrophages into the pro-inflammation phenotype (M1) and amplify the pro-inflammatory phenotype of M1 macrophages via increase of *C-jun*. Therefore, we put forward a hypothesis that M1 macrophages enriched in the high-risk group had a certain association with hypoxia and *JUN*, which was highly expressed in the high-risk groups. Although activated memory CD4+ T cells were significantly different in three datasets, the difference of activated memory CD4+ T cell infiltration in both risk groups was inconsistent. In TCGA and GSE32548, the high-risk group had higher activated memory CD4+ T cells, while activated memory CD4+ T cells presented an opposite trend in GSE13507 (Figure 9). As for the inconsistence, perhaps more real-world experiments were needed to uncover the relationship between hypoxia and activated memory CD4+ T cells.

Lots of researches have demonstrated that tumor mutational burden was closely related to the immunotherapy effect (Hugo et al., 2017; June et al., 2018). The more mutational genes existed in tumor cells, the more mutation-associated RNAs and proteins might be generated, which could be recognized and targeted by the immune system (Rizvi et al., 2015). Therefore, a high mutation burden in tumors was associated with improved immunotherapy response, durable clinical benefit, and progression-free survival (Rizvi et al., 2015). In our study, we listed the top 20 mutational genes in the low- and high-risk groups and then compared risk scores between the wild types and the mutation types of top mutational genes. Finally, we found that the risk scores in the wild type of *MUC16* and *FGFR3* were significantly higher than those in the mutation types of *MUC16* and *FGFR3*, while the risk scores in the mutational *TP53* and *RB1* were higher than those in the wild type (Figure 10). *TP53* was a cancer-suppressor gene, which encoded p53 protein involved in regulating many target genes. Mutations in *TP53* were frequently observed in cancers and were closely related with prognosis of patients with bladder cancer (Ciccarese et al., 2017; Wu et al., 2019). To date, it has become an attractive therapy that restoring functional p53 protein in cancer cells by small peptide molecules (Ciccarese et al., 2017). Similarly, *RB1* is also a cancer-suppressive gene and its mutation is common in bladder cancer samples (Felsenstein and Theodorescu, 2018). Choi once reported that bladder cancer with mutation of *RB1* and *NFE2L2* can be enriched into basal subtype of muscle-invasive bladder cancer (Choi et al., 2017), which could be divided into epithelial–basal and more clinically aggressive mesenchymal subtypes (Guo et al., 2019). Cotton et al. (2017) once screened prognostic biomarkers and identified *MUC16* as a poor prognostic biomarker in advanced-stage bladder tumors. *FGFR3* was a carcinogenic driver, and the mutation, activation, and overexpression of *FGFR3* are common in bladder cancer (Guancial et al., 2014; Pouessel et al., 2016). Ahmad once explored the frequency of *FGFR3* mutation in Indian bladder cancer patients and found that FGFR3 mutations were more common in the low pathological stage and low-grade tumors (Ahmad et al., 2018). Taken together, the effect caused by mutation of *TP53*, *RB1*, *MUC16*, and *FGFR3* were in accord with risks predicted by hypoxia risk scores in our study.

Finally, we enriched different genes in different risk groups and found that cancer- and immune-associated pathways were enriched in the high-risk group, while the metabolism-related pathway belonged to the low-risk group. The cancer-associated pathways were enriched in the high-risk group, which might imply the poor prognosis of patients with high risk scores. Meanwhile, the immune-related pathways in the high-risk groups included autoimmune disease pathways and inflammatory pathways, which was not related to immune response or immune-suppressive pathways. Bladder cancer patients with higher hypoxia risk scores had higher IC50 of chemotherapy drugs in the three independent cohorts, which seemed to be in accordance with the current opinion that hypoxia in the tumor microenvironment played significant roles in treatment resistance.

This is the first hypoxia-related signature in bladder cancer, which could accurately predict the survival outcomes in patients with bladder cancer compared with the traditional pathological parameters. Moreover, the molecular signature has close relationships with clinical–pathological parameters, some infiltrating immune cells, and mutational genes in tumors. As for the stability and practicability of our signature, it still needs to be validated in future clinical practices.

## Data Availability Statement

The original contributions presented in the study are included in the article/Supplementary Material, further inquiries can be directed to the corresponding author/s.

## Ethics Statement

Ethical review and approval was not required for the study on human participants in accordance with the Local Legislation and Institutional Requirements. Written informed consent for participation was not required for this study in accordance with the National Legislation and the Institutional Requirements.

## Author Contributions

FZ and XW designed the study. HH, DL, and YB collected the transcriptome and clinical data. YB, YY, HH, JW, YT, HM, and DF took part in the data collection. FZ drafted the manuscript. PH revised the final manuscript. All authors contributed to the article and approved the submitted version.

## Conflict of Interest

The authors declare that the research was conducted in the absence of any commercial or financial relationships that could be construed as a potential conflict of interest.

## Footnotes

1 https://www.cancerrxgene.org

2 http://www.r-project.org/
