# Supplementary material for: Development and Validation of a Hypoxia-Related Signature for Predicting Survival Outcomes in Patients With Bladder Cancer
Source: Front Genet. 2021 May 26;12:670384. doi: 10.3389/fgene.2021.670384 (PMC8188560; doi:10.3389/fgene.2021.670384)
Supplement: Supplementary file 1 [file Presentation_1.pdf]

Supplementary Material

Supplementary Figures

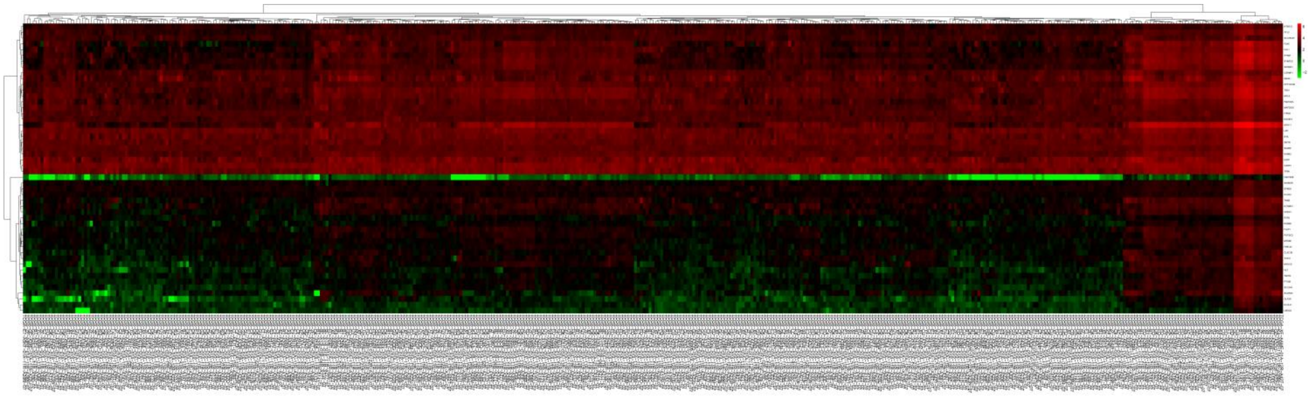

Supplementary Figure 1. Selection of differentially expressed genes in TCGA dataset with FDR<0.05 and |log Fold-Change|⩾1. The heatmap listed top 50 differentially expressed genes.

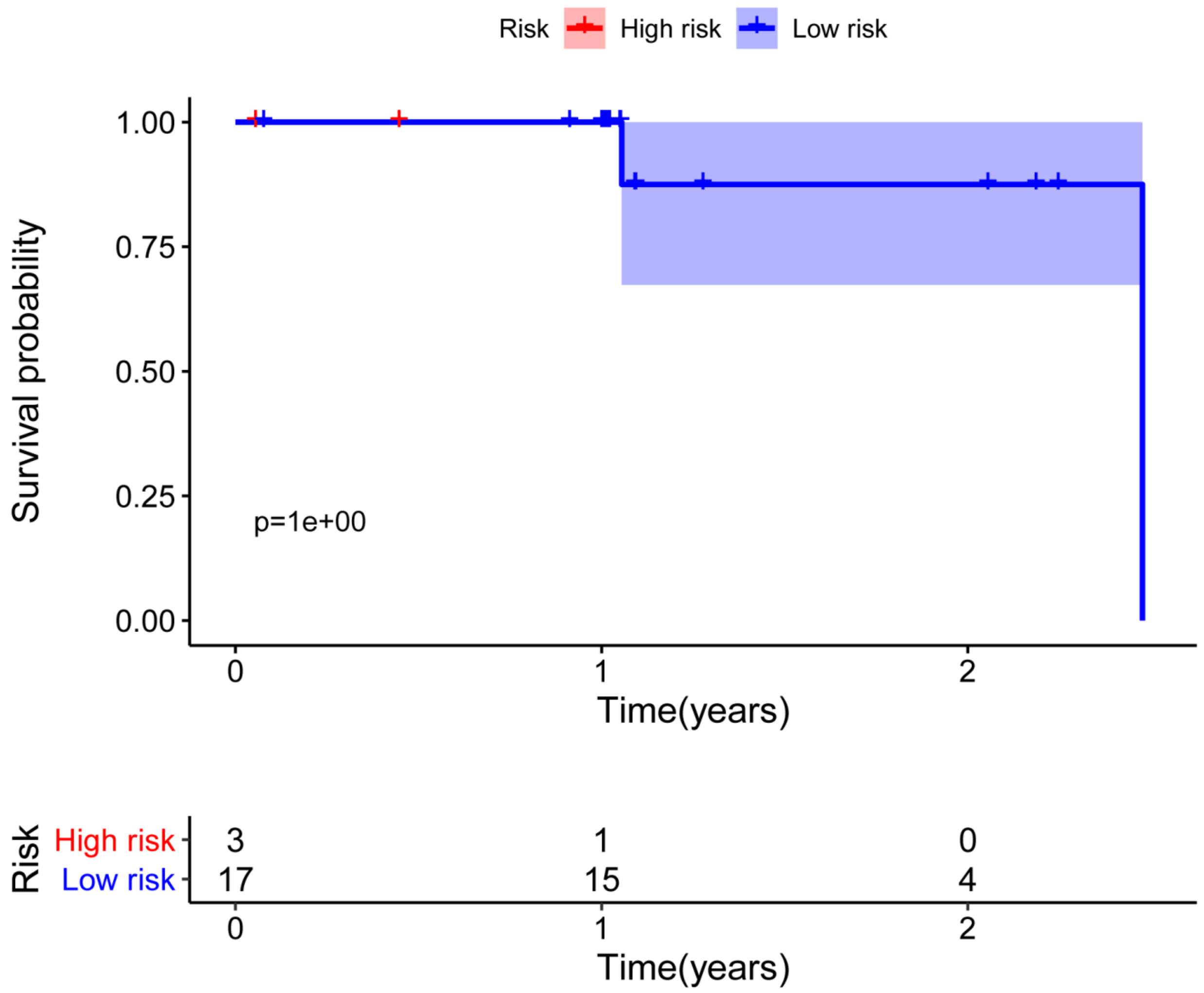

Supplementary Figure 2. Kaplan-Meier curves showed the patients in the subgroup of low pathological grade.

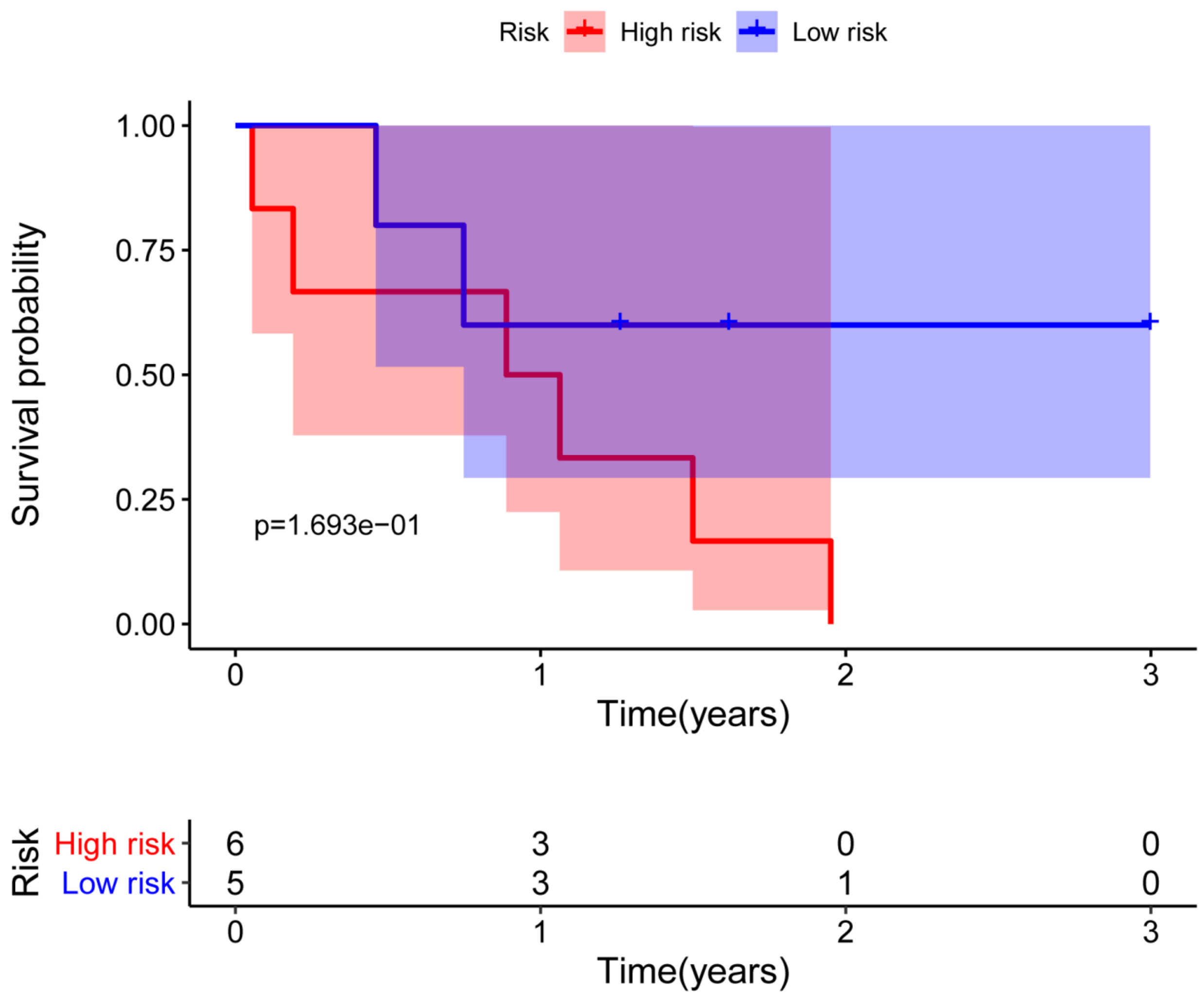

Supplementary Figure 3. Kaplan-Meier curves showed the patients in the subgroup of metastasis.
